# Prevalence and causes of low vision and blindness in Baotou: A cross-sectional study

**DOI:** 10.1097/MD.0000000000004905

**Published:** 2016-09-16

**Authors:** Guisen Zhang, Yan Li, Xuelong Teng, Qiang Wu, Hui Gong, Fengmei Ren, Yuxia Guo, Lei Liu, Han Zhang

**Affiliations:** a Department of Ophthalmology, Hohhot Chao Ju Eye Hospital, Hohhot; b Department of English, School of Fundamental Sciences, China Medical University, Shenyang; c Department of Ophthalmology, Hebei Xinji Fuming Eye Hospital, Shijiazhuang; d Department of Ophthalmology, Baotou Chao Ju Eye Hospital, Baotou; e Department of Ophthalmology, The First Affiliated Hospital, China Medical University, Shenyang, China.

**Keywords:** blindness, low vision, prevalence

## Abstract

The aim of this study was to investigate the prevalence and causes of low vision and blindness in Baotou, Inner Mongolia.

A cross-sectional study was carried out. Multistage sampling was used to select samples. The visual acuity was estimated using LogMAR and corrected by pinhole as best-corrected visual acuity.

There were 7000 samples selected and 5770 subjects included in this investigation. The overall bilateral prevalence rates of low vision and blindness were 3.66% (95% CI: 3.17–4.14) and 0.99% (95% CI: 0.73–1.24), respectively. The prevalence of bilateral low vision, blindness, and visual impairment increased with age and decreased with education level. The main leading cause of low vision and blindness was cataract. Diabetic retinopathy and age-related macular degeneration were found to be the second leading causes of blindness in Baotou.

The low vision and blindness were more prevalent in elderly people and subjects with low education level in Baotou. Cataract was the main cause for visual impairment and more attention should be paid to fundus diseases. In order to prevent blindness, much more eye care programs should be established.

## 1 Introduction

With the rapid economic development, the problem of an aging population is seriously. Low vision and blindness have been very important public health problems worldwide.^[1]^ In 1999, the World Health Organization proposed “Vision 2020: The Right to Sight” which has contributed to a reduction in the number of cases of blindness in the world.^[2]^ Previous report by The Vision Loss Expert Group of the Global Burden of Disease Study showed that the prevalence of visual impairment was about 10% in adults aged 50 years or older in China.^[3]^ In the rural Chinese population in Kailu which is a county in Tongliao city of Inner Mongolia, the overall prevalence rates of visual impairment and blindness using presenting visual acuity (PVA) were 9.8% and 2.2%, respectively, and were adjusted to 4.7% and 0.9% based on the best-corrected visual acuity (BCVA), respectively.^[4]^ Kailu is in the east of Inner Mongolia, and most of the population is rural. However, Baotou (40°38′00″N, 109°59′00″E) is the biggest City of the Inner Mongolia. Baotou spans about 27,768 km^2^ and had a population of about 2.76 million in 2013. The majority of the population in the region is Han Chinese, with a substantial Mongol minority. In Baotou, the main religion is Tibetan Buddhism, and most residents are herdsmen. Climate in Baotou is very different during the year. Winter is cold and can be very long, with frequent blizzards. Usually summer is short and warm. There are some vast grasslands and deserts in Baotou, so the geography is different from Kailu. Understanding the epidemiology of low vision and blindness in Baotou population could help the government to make policy to prevent blindness in Inner Mongolia. To date, there is no report about the prevalence of low vision and blindness in the Inner Mongolia population, both in rural and urban. The aim of this cross-sectional study was to investigate the prevalence and causes of low vision and blindness in Baotou city located in the middle of Inner Mongolia Autonomous Region of China based on age, sex, area, and education.

## 2 Methods

### 2.1 Subjects

According to multistage sampling, there were 8 regions in this study including 5 regions in urban (Rare Earth High-Tech Industrial Development sub-district, Tiexi sub-district, Anshan Dao sub-district, Qianjin Dao sub-district, and Gaoyoufang community) and 3 regions in rural (Bailingmiao town, Haizi village, and Yinhao town) area with a sample of 7200 people 40 years of age or older (900 people in each region). The list of names of residents was obtained from the village or community registers. Through door-to-door home visits or telephone, there were 5770 residents enrolled in this study (response rate: 80.14%). This investigation was conducted from January 2013 to November 2013. The ethnicity includes Han, Hui, Mongolia, and Man groups.

This population-based cross-sectional study was approved by the Chao Ju eye hospital ethics committee and adhered to the Declaration of Helsinki. Written informed consent was obtained from all participants during the questionnaire stage.

### 2.2 Examination

Questionnaire was carried by investigators. The information extracted from the questionnaire records of all subjects included age, sex, education level, area, and eye disease history. The causes of low vision and blindness were determined by well-trained ophthalmologists. Two well-trained ophthalmologists tested PVA separately in each eye using a “standard logarithmic visual acuity chart” at a distance of 5 m. If the person was wearing glasses, then PVA was recorded with glasses. If his/her PVA was <20/60 in either eye, pinhole visual acuity was assessed. All participants underwent basic eye examination including cornea, anterior chamber, lens using the slit-lamp biomicroscopy (Slit Lamp, Six-six, Suzhou, China), and fundus using direct ophthalmoscopy and Non-Mydriatic Fundus Camera (NW-300, Topcon, Japan). The causes of visual impairment/low vision/blindness were according to the examining ophthalmologist including an 8-item list (refractive error, cataract, aphakia eye, corneal opacity, glaucoma, age-related macular degeneration (AMD), diabetic retinopathy (DR), and other cause). After the eye examination, the subjects with ocular diseases were advised for further treatment in hospital.

### 2.3 Definition

In our study, visual impairment was defined according to the WHO definition, and was reported based on the BCVA using pinhole.^[5]^ Visual impairment includes low vision and blindness. Low vision was defined as visual acuity (VA) in the better eye of 20/400–20/60. Blindness was considered as VA in the better eye <20/400. If the subject had different cause for visual impairment or if each eye had more than one causes, the one that was more correctable was defined as the cause of visual impairment.^[6,7]^

### 2.4 Statistical analysis

Data were entered into a database in Microsoft Excel (2003). The prevalence of low vision, blindness, and visual impairment was calculated as percent with 95% confidence interval (CI). Odds ratio was calculated using χ^2^ test. Statistical analyses were performed using SPSS V.20.0 (SPSS Inc., Chicago, IL).

## 3 Results

**Figure 1:**
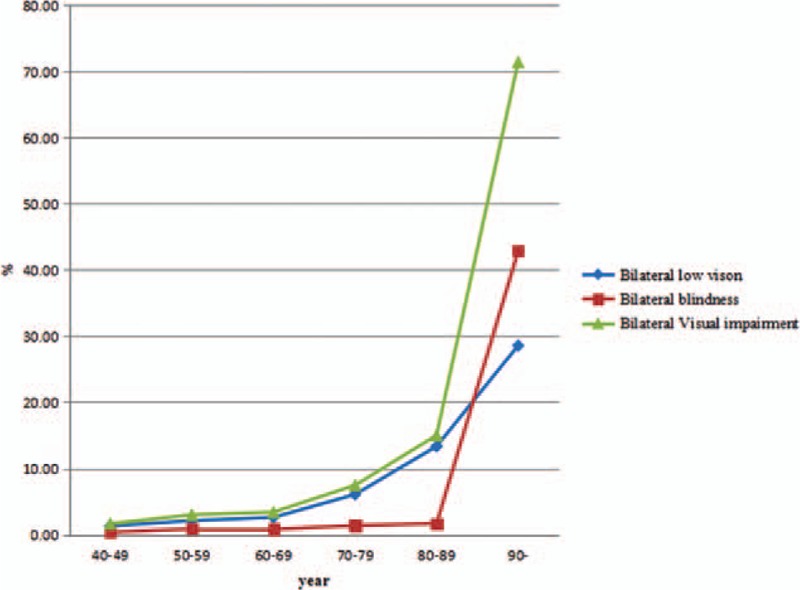
Prevalence of bilateral low vision, blindness, and visual impairment by age group.

**Figure 2:**
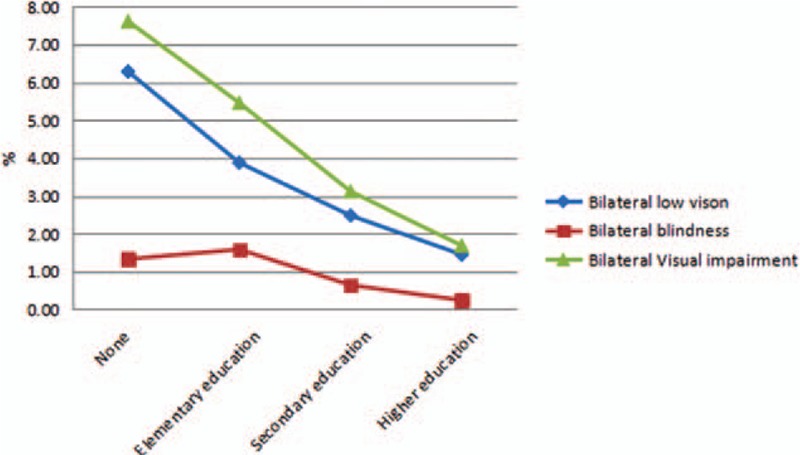
Prevalence of bilateral low vision, blindness, and visual impairment by education level.

A total of 5770 individuals were recruited in Baotou. The characteristics of the subjects in this study are shown in Table 1. The average age of all subjects was 59.2 ± 8.6 years. The overall bilateral prevalence rates of low vision and blindness were 3.66% (95% CI: 3.17–4.14) and 0.99% (95% CI: 0.73–1.24), respectively. In addition, the prevalence rate of bilateral visual impairment was 4.64% (95% CI: 4.1–5.19). Table 2 shows that there was no statistical significance of prevalence rate of bilateral low vision and blindness between male and female. In area aspect, there was no statistical significance of prevalence rate of bilateral low vision and blindness between urban and rural population. The prevalence of bilateral low vision, blindness, and visual impairment increased with age (Fig. 1) and decreased with education level (Fig. 2). Table 3 shows that the prevalence of low vision and blindness increased with age and decreased with education level, including right eye, left eye, and monocular. However, there was no statistical significance of prevalence of unilateral low vision and blindness between different sex and area. A total of 211 subjects had low vision (Table 4). The top 4 causes of low vision were cataract, uncorrected refractive error, glaucoma, and DR. There were 57 subjects who were blind (Table 5). The leading cause of blindness was cataract [31 (54.39%)]. DR [6 (10.52%)] and AMD [6 (10.52%)] were found to be the second leading causes of blindness in Baotou.

**Table 1:**
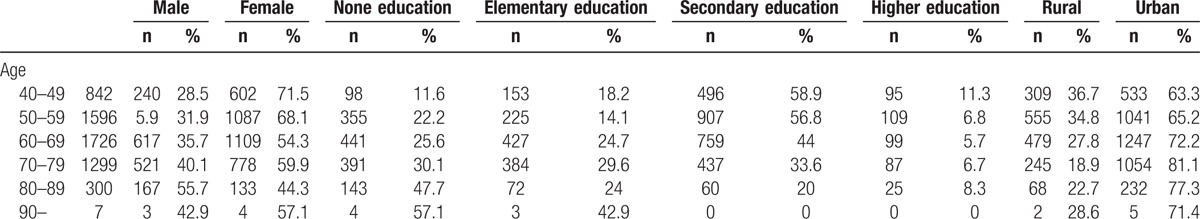
The characteristics of subjects.

**Table 2:**
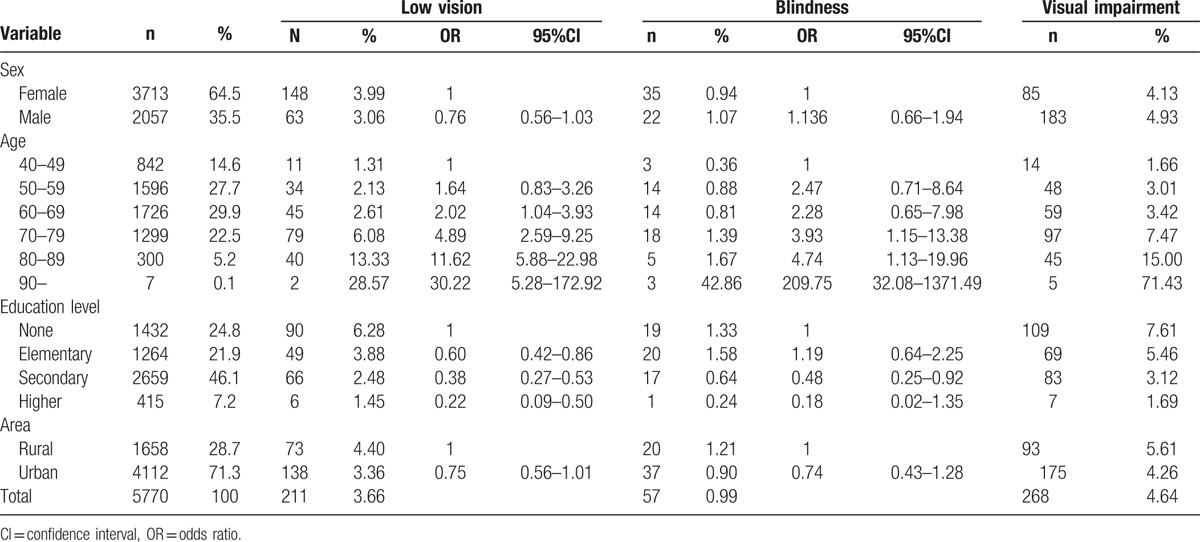
The prevalence of bilateral low vision, blindness, and visual impairment.

**Table 3:**
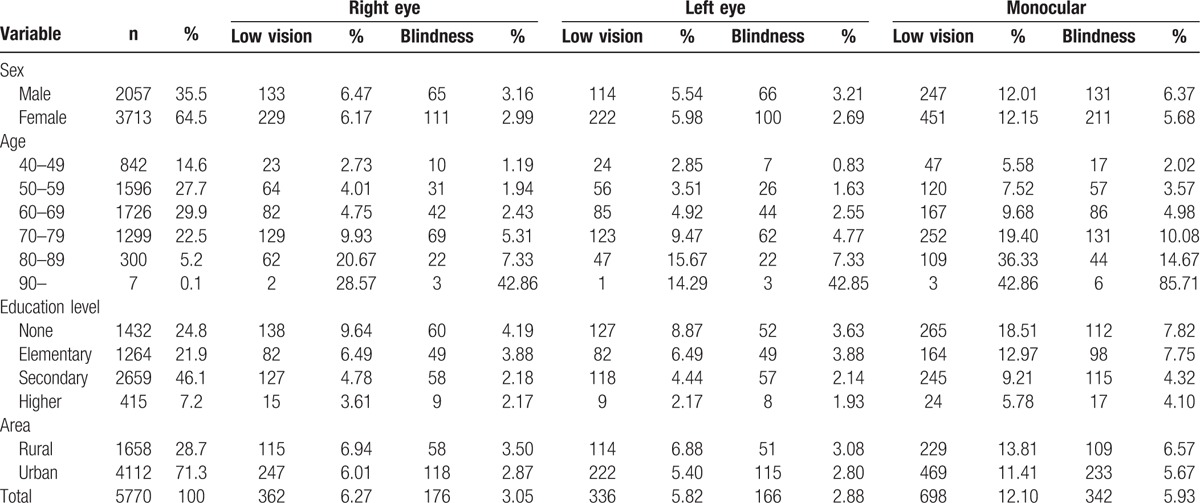
The prevalence of unilateral low vision and blindness.

**Table 4:**
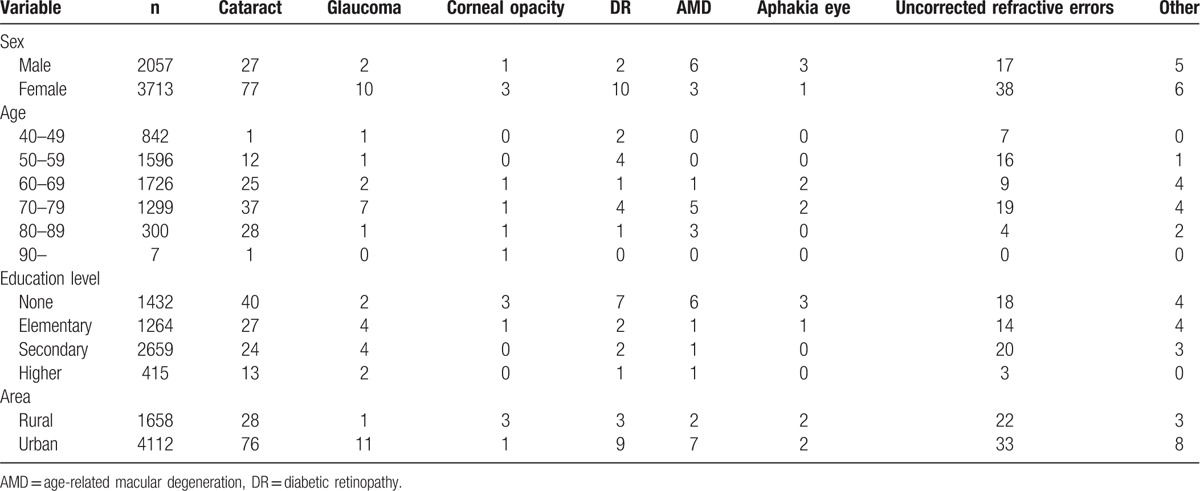
Causes of low vision in Baotou.

**Table 5:**
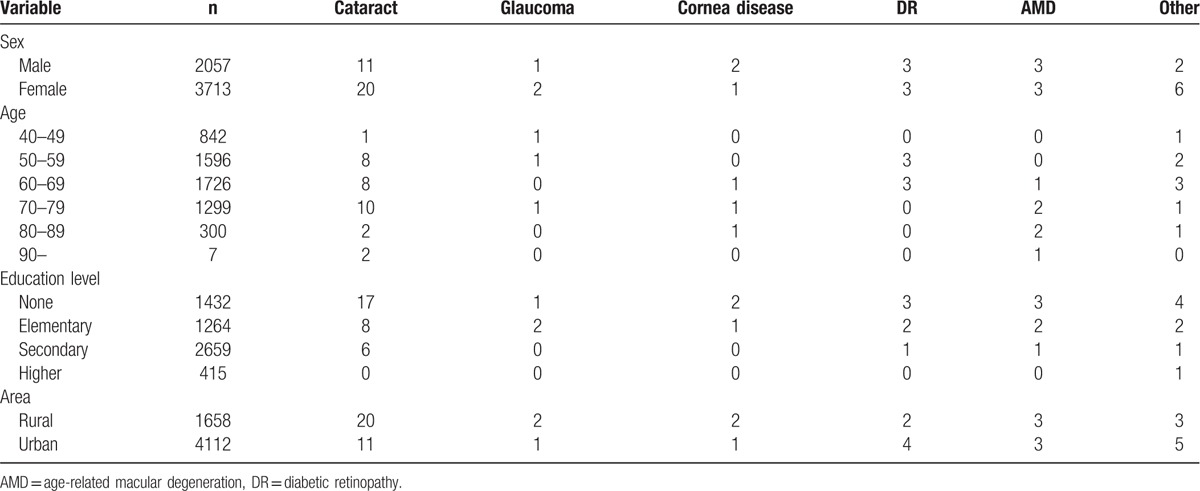
Causes of blindness in Baotou.

## 4 Discussion

To the best of our knowledge, this was the first study in Baotou (middle of Inner Mongolia) to investigate the prevalence of low vision, blindness, and visual impairment. A meta-analysis including 24 population-based studies by Chen et al reported that the prevalence rates of blindness and low vision were 1.7% and 4.1%, respectively, among the older adults over 50 years of age in mainland China till 2010.^[8]^ The studies estimating the prevalence of blindness and low vision in China published from January 2010 to April 2016 were searched and presented in Table 6.^[4,9–20]^ Although the estimating criteria for blindness and low vision differed among studies, Table 6 shows that according to the BCVA in better eye, previous studies reported that the prevalence rates of blindness and low vision were 0.2% to 2.88% and 1.4% to 10.41% in China, respectively. In rural of Kailu County, Inner Mongolia, the overall prevalence rates of blindness and low vision based on BCVA were 0.9% and 7.52%, respectively. The prevalence of blindness was lower than that in Baotou (1.21%), but the prevalence of low vision was higher than that in Baotou (4.4%).

**Table 6:**
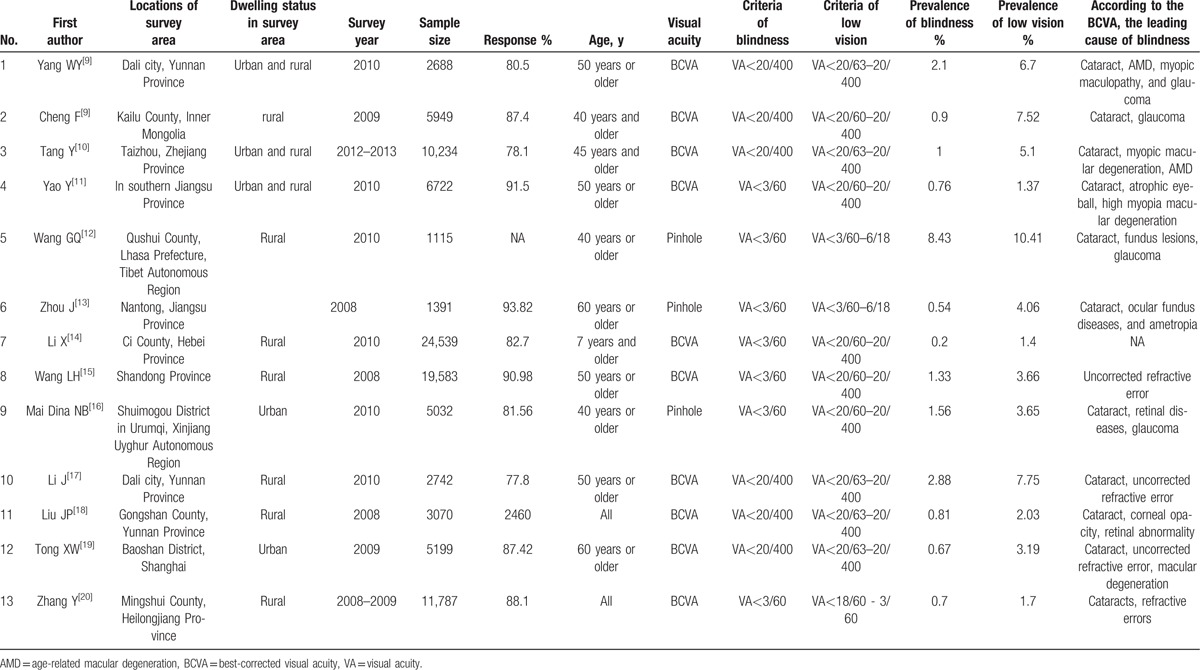
The studies estimating the prevalence of blindness and low vision in China published from January 2010 to April 2016.

According to the results of this investigation, the prevalence of low vision and blindness increased with age and decreased with education level in Baotou. The prevalence of blindness and visual impairment in Qidong City of Jiangsu Province were higher in women and aged, illiterate persons.^[21]^ This result was in accordance with most study results. So, more attention should be paid to the eye health of elderly subjects or residents with low education level. In addition, the prevalence rate of visual impairment in urban population and rural population were 1.40% and 2.22%, respectively.^[22]^ However, no sex and area difference was seen in our study.

The main cause of low vision and blindness was cataract according to the BCVA. However, the cause of low vision and blindness was uncorrected refractive error in Shandong. In this study, the top 4 causes of low vision were cataract, uncorrected refractive error, glaucoma, and DR. In addition, the leading cause of blindness was cataract. Fundus diseases (DR and AMD) were found to be the second leading causes of blindness in Baotou.

WHO reported that the main cause of blindness was cataract (51%). The other causes included glaucoma, AMD, childhood blindness, corneal opacities, uncorrected refractive errors, trachoma, and DR.^[23]^ This was same as the results of present investigation. Many programs have been carried out in China to avoid cataract blindness such as “Million Cataract Surgeries Program” (MCSP), but cataract was always the main cause of blindness. In Guangdong and Shanghai rural hospitals, the cataract surgical rate (CSR) was 643 and 532.^[24]^ However, the CSR in urban districts of Shanghai was 5468 in 2012.^[25]^ In order to prevent the main cause of blindness, most cataract screening and operations are needed to be done in China.

There are some limitations to this study. First, the prevalence rates of low vision and blindness were based on BCVA using pinhole rather than PVA. So the results lacked prevalence of low vision and blindness caused by uncorrected refractive error. Second, serious cataract and corneal opacity may affect detecting fundus diseases such as DR, AMD, and so on.

In conclusion, the overall bilateral prevalence rates of low vision and blindness were 3.66% and 0.99%, respectively. The prevalence rates of low vision and blindness were associated with age and education level. More attention should be paid to the eye health of elderly people and people with low education.

## Acknowledgments

We thank the Community Committee of Rare Earth High-Tech Industrial Development Region, Kundulun District, Jiuyuan District, Donghe District, Baiyun Obo Mining Region, Tumote-You County, Damao County, and Guyang County.
